# Negative effects of COVID-19 pandemic on adolescent health: Insights, perspectives, and recommendations

**DOI:** 10.7189/jogh.12.03009

**Published:** 2022-05-21

**Authors:** Aparajita Ashwin, Sathya D Cherukuri, Ashwin Rammohan

**Affiliations:** 1 Wellington School, Wellington, UK; 2 Somerset NHS Foundation Trust, Taunton, UK; 3 The Institute of Liver Disease & Transplantation, Dr. Rela Institute & Medical Centre, Bharath Institute of Higher Education & Research, Chennai, India

The adverse mental health effects of the COVID-19 pandemic and its control measures (lockdowns, quarantine, etc.) among the adult population have been widely acknowledged [1]. There are, however, only a few studies on the topic focused on children and adolescents under the age of 18 [2]. Children constitute a third of the global population and those aged between 10 to 18 years make up approximately a fifth [3]. Although children are less likely to be affected by COVID-19, they are not indifferent to the detrimental effects that the pandemic and the lockdown could bring upon their mental health.

There remains a lack of data on the true impact of this pandemic on children, especially adolescents. Research has been limited, and at times inconsistent [2,4-6]. It has been suggested that compared to adults, the pandemic may have worse long-term physical, psychological, educational, and social consequences for children and adolescents [2,4-6]. We attempted to provide insights into the mental health consequences of the pandemic on this subset of the population from the perspectives of a young adult, a parent, and a psychiatrist.

## EFFECT OF THE PANDEMIC ON YOUNG ADULTS

Adolescence is a fragile period, marked by rapid biological and social changes, and common mental disorders are known to manifest during this time. Young adults are particularly susceptible to the vagaries of a pandemic due to a limited grasp of its intensity and inadequate coping mechanisms [5]. Studies have unequivocally shown that disruptions to routines, amplified family stressors, social isolation, and domestic violence create an environment that exponentially increases adolescent psychiatric issues. Thus, while superficially appearing to be physically less vulnerable, young adults can experience significant psychological difficulties due to this global health event [2,4,5,7].

Schools introduce children to new ideas, teach them to socialize, make friends, and participate in activities that enhance growth. Educational systems not only provide for academic learning, they serve as platforms for other social-support initiatives like counselling services, after-school clubs, or health provisions like vaccination clinics and nutrition programs. Additionally, these institutions help mitigate the effects of inequalities among socio-economically disadvantaged and marginal members of society. Facility closures, social isolation, and quarantine have caused a loss of social connection with teachers, friends, and peers. Decreased physical activity, loss of tutor time, and increased ‘screen time’ through virtual learning, social or digital media can adversely affect mental health. Students who rely on special education, lack digital access or tools, or live in unstable home settings risk falling behind their peers as schools move online and have decreased peer contact. Further consequences of this increased digital presence include disturbance of the circadian rhythm and increased susceptibility to bullying and abuse. Social media can heighten stress, exacerbate, or trigger psychiatric issues. However, social media may also help as a coping mechanism, working as an outlet for adolescents by increasing social interactions, self-expression, and providing for easy access to information.

**Figure:**
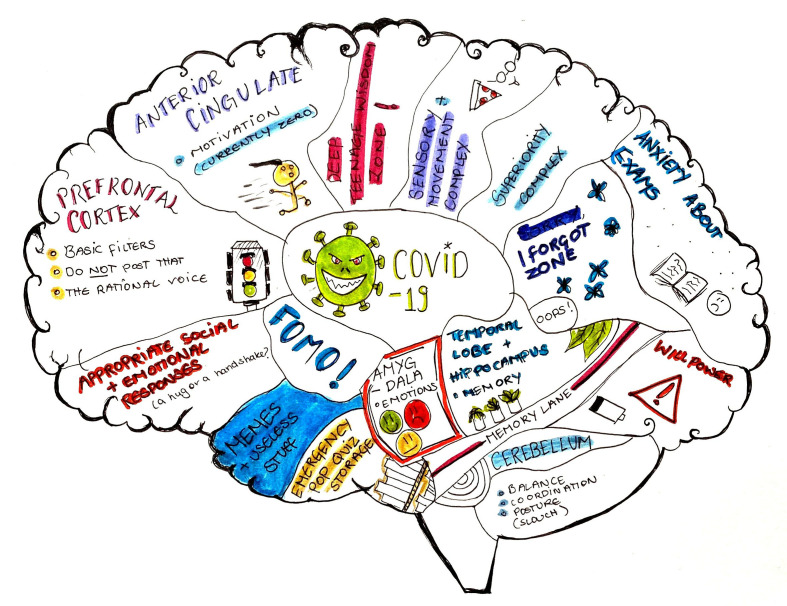
Photo: Insights into an adolescent mind through the pandemic. From the first author’s personal collection, used with permission.

Other stressors include separation, loss, and grief due to separation from primary caregivers owing to illness, lockdown or death. Anxiety or fear of their own or their parent’s death is a natural response in young adults. This becomes magnified when the caregivers are frontline workers at an increased risk of COVID-19. Companionship is an essential aspect of normal psychological development and well-being. Separation from caregivers pushes children into a state of crisis and increases the risk of psychiatric disorders. Several studies from the current pandemic suggest that children quarantined under the suspicion of having COVID-19 or testing positive for the disease are more likely to develop mental health disorders such as acute stress, anxiety, and adjustment disorders [8-10]. Furthermore, a study showed that children who had COVID-19 or were quarantined had a four times higher likelihood of developing posttraumatic stress [11]. Children with special needs are particularly vulnerable to the detrimental psychological impacts of this pandemic. Those with autism and neurocognitive disability may feel frustrated due to disruptions in their daily lives.

Adolescents can sense their parents’ stress and often show their worries in ways that adults may interpret as temper tantrums. Parents may notice that their children have become fussy and/or more aggressive. These reactions in children in turn have a domino effect on the parents, leading to uncertainty, difficulties in empathizing, a sense of sadness, anxiety, feeling of lack of control, as well as sleep deprivation. Adolescents also have amplified energy levels, novelty, motivation, curiosity, and enthusiasm, making it difficult for them to isolate socially. Important events during this stage of psychological development include maintaining social relationships. Adolescents may feel upset missing out on peer-group related social activities like sports, parties, social gatherings, etc. Adolescence-associated hormonal changes, combined with age-related social dynamics make young adults attuned to social status, peer groups, and relationships. Adolescents may feel as though they are lonely, frustrated, nervous, or missing out due to social distancing during the pandemic. Fears regarding the future among adolescents are often expressed as sadness, anger, disillusionment, withdrawal, and/or depression (**Table 1**), all of which lead to reduced life satisfaction, including fear of death, infection, and the unknown. Other presentations include diminished interest in social activities, peers, and school. The affected adolescent may also show increased risk-taking behaviour in the form of failing to follow social distancing protocols, alcohol or substance abuse, or increase in self-harm as a means of coping. They may also express disillusionment and concerns regarding faith in governmental policies, institutions and justice.

**Table 1:** Physiological behavioural responses to the pandemic, practical guidance for the caregiver and when to refer for specialist mental health assessment

**A. Normal adolescent behavioural and functional responses to the pandemic**
• Able to understand many of the implications
• Legitimate fears about the future (eg, economic repercussions, health issues)
• Varied responses: worry, fear, sadness, anger, disillusionment, avoidance, withdrawal
• Anxiety, eg, “Will I be okay?”, “Will my parents be okay?”, “Will I ever see my friends again?”, “Will my school ever open again?”, “What will my life be like?”
• Depressive symptoms such as loss of hope and future orientation: “Nothing will ever get better.”
• Reduced life satisfaction
• Fearfulness, eg, of illness, death, the unknown
• Sleep disturbances related to anxiety, depression, and disruption of routines
• Decreased interest in social activities, peers, school
• Increased risk-taking behaviour: failing to wear mask or social distance, potential alcohol or other substance abuse, unsafe sexual behaviours
• Difficulty concentrating and, academic difficulties
• Concerns about public institutions, justice, power, and control
• Desire to discuss the policy issues involved
• Able to integrate multiple factors in understanding illness and alternative possibilities
**B. Practical guidance for parents & caregivers**
• Show respect for their feelings
• Discuss their opinions, feelings, and different ways to cope
• Discuss causes and effects of the COVID-19 pandemic
• Listen to and talk about what is present in social media
• Be present if they watch television; discuss and provide perspectives
• Check in regularly
• Address risk taking behaviours clearly and directly
• Share and talk about reactions to COVID-19 related disruptions (eg, virtual learning, etc)
• Normalise experiences: “You’re not alone; everyone is struggling”
• Encourage connection with friends—virtually or with advised safety precautions
• Be a role model: Share your own feelings and coping mechanisms
• Help them engage in community or other projects as appropriate
**C. Symptoms which require mental health referral**
• Anxiety and/or depressive symptoms
• Increased arousal, mood changes, irritability, withdrawal, emotional numbing, being overwhelmed
• Physical symptoms such as fatigue, headaches, or aches that cannot be medically explained
• Disordered eating habits
• Sleep disturbances, including unrestful sleep and trouble falling asleep
• Traumatic grief
• Symptoms of posttraumatic stress disorder—eg, that disrupt functioning and/or can create risk of harm to self or others
- Nightmares
- Re-experiencing the event/disaster
- Intrusive thoughts that interfere with focus, concentration and attention
- Increased arousal – may lead to aggressive behaviour
- Hypervigilance
- Avoidance of activities, experiences, or places associated with the event
- General withdrawal
- Emotional dysregulation or dissociation
**D. Symptoms which require emergency mental health referral**
• Suicidal ideation, intent, plan or attempt
• First known self-cutting
• Intense fear, anxiety, helplessness, panic or horror,
• Presence of dissociative symptoms such as detachment and depersonalisation
• Extreme confusion or inability to make simple decisions
• Uncontrollable and intense grief
• Intrusive thoughts or severe cognitive impairment
• Debilitating physical complaints in the absence of medical explanation

Although there are no long-term outcome studies on the adverse effects of the COVID-19 pandemic on adolescent mental health, extrapolation from other pandemics and disasters suggest that these young adults are likely to have long-term adverse effects of escalated risk of mood disorders, psychosis, and death by suicide in adulthood [9,12-18]. Furthermore, social isolation measures and loneliness have shown to increase the risk of depression up to 9 years later. The duration rather than intensity of loneliness and the presence of previous mental health disorders are strong risk factors for these mental health symptoms [9,13-16].

Nonetheless, differentiating psychiatric ailments from a normal stress response requires a high index of suspicion. Anxiety and depression may be masked as increased mood swings, irritability, withdrawal, and emotional dysregulation [2,19]. Physical symptoms such as fatigue, headaches, and others that cannot be medically explained, including those of disordered eating habits and self-harm, are not uncommon. Intense fear, panic, or anxiety that disrupts basic functioning such as sleeping, eating, family and peer interactions, etc. are warning signs. Confusion or the inability to make simple decisions, uncontrollable grief, intrusive thoughts, or severe cognitive impairment immediately require professional help [2,6,19].

## EFFECT OF THE PANDEMIC ON FAMILIES

School closures and the hiatus of after-school activities has added to parental pressure to balance responsibilities, including becoming the sole providers of supervision and education for their children – all while experiencing heightened stresses of financial instability, social isolation, boredom, frustration, fear of infection, and changes to health and social care access. A large Canadian cross-sectional study showed that, when compared to parents without children, a significantly higher number of parents with children at home reported deterioration of mental health (35 vs 44%) during this pandemic [7]. This cohort was also found to have a higher incidence of suicidal ideation and alcohol abuse. A collusion of these factors has resulted in a surge in the incidence of domestic violence, and physical punishment of children. Parents reported more adverse interactions with their children, including more yelling and conflicts, further reinforcing the negative mental health impact of the social isolation on these young adults.

Remarkably, a nationwide survey noted that some parents reported increased positive interaction with their children, including spending more quality time (65.4%), feeling closeness (49.7%), and showing love (44.5%) [7]. Furthermore, previous research from other epidemics has also shown that while parenting pressures increase, so do opportunities to strengthen family bonds [5].

## RECOMMENDATIONS

**Figure 1:**
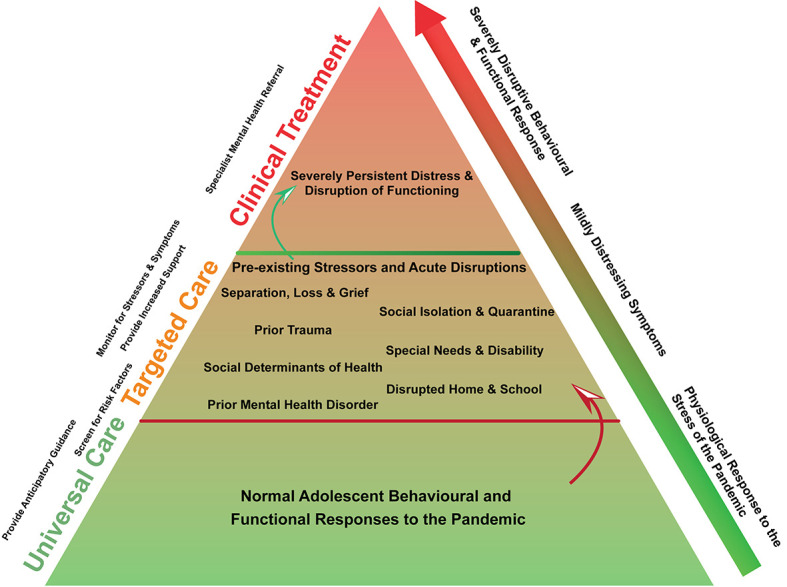
A stepwise multi-tiered model for support and management of adolescent mental health during the COVID-19 pandemic.

With health services being overwhelmed by the sheer magnitude of the pandemic, delegation of personnel towards nurturing mental health remains a challenge. It is therefore essential to enable and utilise our inherent coping mechanisms to the fullest (**Table 1****,** **Figure 1**).

### Improve communication, validate feelings & tackle sensitive issues

Simple interventions such as showing respect for the adolescent’s feelings and validating their fear allows for a much more open and transparent channel of communication. Cross-sectional surveys highlight the importance of communication in mitigating symptoms of anxiety and depression [5,7,11]. It is imperative that their questions are answered directly and honestly, providing them with information in an open, age-appropriate, reassuring manner. It is also important to debate and explain what is being presented on television and social media. Discussing the pandemic, addressing the disruptions, and encouraging participation in the family’s decision-making helps young adults feel validated and empowered. It builds an implicit belief that an adult is always available.

Talking about solemn topics like illness and death needs to be done by giving simple, clear information about family health problems, in calm and neutral tones, while avoiding minimisation of serious health threats. It is vital to provide realistic reassurance and avoid false promises that might later damage the child’s ability to trust (eg, “Don’t worry, you will be fine”). Normalising experience, reinforcing the fact that they are not alone in this fight, sharing parental feelings and coping mechanism along with keeping routines and focusing on positive behaviour nurtures a calming environment.

### Bolster parental mental well-being

Supporting parental health is also extremely important due to its tumble-down effect on the younger members of the family. Strong family relationships and positive interactions are protective considerations which can reinforce resilience. It is important for other adult family members to appreciate parents’ efforts. Reinforcing positive coping and stress reduction strategies like physical activity, regular use of green spaces, and pursuing safe social connections and routines helps promote parental and child health. These techniques have shown to dramatically reduce hypervigilance, lack of trust in adults, self-regulation issues, and inappropriate social interactions among adolescents.

### Identify vulnerable subsets of adolescents

Assigning medical personnel towards mental health screening during these trying times remains a challenge. However, with the waves of COVID-19 pandemic plateauing, mental health screening for its aftermath becomes a realistic proposition. Screening can detect emotional and behavioural functioning/psychosocial symptoms, and help identify when referral for evaluation and treatment are needed. Mental health workers provide crucial support in the form of anticipatory guidance, symptom-specific support, suggested interventions, screening for risk factors, and early escalation of care. Guidance and support will help schools identify and support children and young people for whom lockdown has been particularly challenging in terms of engagement with virtual learning in order to ensure their learning needs are fulfilled.

### Online professional counselling

Telemedicine or eHealth has been an integral part of health care-delivery for over two decades. However, the advantages of digital health resources have been particularly apparent during this pandemic. With social distancing measures in place, digital solutions have provided new ways of medical care services and support. Online therapy sessions or “cyber-counselling” has become a reasonable counselling conveyance strategy during this pandemic [16,18,20,21]. An irrefutable advantage of online counselling is that of availability and reach. It touches populaces that would not immediately look for face-to-face counselling. It also offers a different way of building rapport: instead of meeting in the counsellor's office, the venue of the virtual counselling is one’s home or private space, allowing for more intimacy. Furthermore, due to the anonymity associated with these sessions, the counselees tend to feel less ashamed about their issues, enabling a sense of fulfilment, smoothness, and session profundity [22-25]. Nonetheless, there have been concerns with respect to reduction of visual prompts, failure to intercede in an emergency, and the need of restorative control. Experts have also expressed concerns regarding the moral issues of cyber counselling, including things such as competence, informed consent, privacy, and security.

The goals of these counselling services include providing anticipatory guidance and strategies of coping with the crisis, screening for risk factors, and appropriate timing and deciding on escalation of care. Studies focussing on COVID-19 online counselling programmes have highlighted the uniqueness of these sessions [16,18,20,21]. As compared to longer, more in-depth service, these sessions are significantly shorter with a pandemic-oriented focus. An offshoot of virtual counselling is therapy via text-messaging. Young adults responded favourably as this facility allows a 24-hour emergency counselling service, along with the benefits of anonymity, and the capacity to re-read and alter written explanations.

## CONCLUSIONS

The long-term psychological effects of this COVID-19 pandemic especially on the young minds remain unclear. Additionally, the future of many young people is uncertain due to the interruption of education. While coping strategies from within the family and the community do help minimise the adverse effects of the pandemic on this generation of youth. It is imperative that policy makers, governmental bodies and stakeholders recognise the importance of education and mental health outcomes alongside those of employment and economic revival, and institute measures which will ensure that the “COVID-19 generation” is not disproportionately disadvantaged.

## References

[1] Xiong J Lipsitz O Nasri F Lui LMW Gill H Phan L Impact of COVID-19 pandemic on mental health in the general population: A systematic review. J Affect Disord. 2020;277:55-64. 10.1016/j.jad.2020.08.001 32799105 PMC7413844

[2] Rider EA Ansari E Varrin PH Sparrow J Mental health and wellbeing of children and adolescents during the covid-19 pandemic. BMJ. 2021;374:n1730. 10.1136/bmj.n1730 34429302

[3] World population by age and region 2021 | Statista. Available: https://www.statista.com/statistics/265759/world-population-by-age-and-region/. Accessed: 7 November 2021.

[4] Hafstad GS Augusti EM A lost generation? COVID-19 and adolescent mental health. Lancet Psychiatry. 2021;8:640-1. 10.1016/S2215-0366(21)00179-6 34090583 PMC9764868

[5] Meherali S Punjani N Louie-Poon S Abdul Rahim K Das JK Salam RA Mental Health of Children and Adolescents Amidst COVID-19 and Past Pandemics: A Rapid Systematic Review. Int J Environ Res Public Health. 2021;18:3432. 10.3390/ijerph18073432 33810225 PMC8038056

[6] Ford T John A Gunnell D Mental health of children and young people during pandemic. BMJ. 2021;372:n614. 10.1136/bmj.n614 33692087

[7] Gadermann AC Thomson KC Richardson CG Gagné M McAuliffe C Hirani S Examining the impacts of the COVID-19 pandemic on family mental health in Canada: findings from a national cross-sectional study. BMJ Open. 2021;11:e042871. 10.1136/bmjopen-2020-042871 33436472 PMC7804831

[8] Loades ME Chatburn E Higson-Sweeney N Reynolds S Shafran R Brigden A Rapid Systematic Review: The Impact of Social Isolation and Loneliness on the Mental Health of Children and Adolescents in the Context of COVID-19. J Am Acad Child Adolesc Psychiatry. 2020;59:1218-39.e3. 10.1016/j.jaac.2020.05.009 32504808 PMC7267797

[9] Orgilés M Morales A Delvecchio E Mazzeschi C Espada JP Immediate Psychological Effects of the COVID-19 Quarantine in Youth From Italy and Spain. Front Psychol. 2020;11:579038. 10.3389/fpsyg.2020.579038 33240167 PMC7677301

[10] Liu JJ Bao Y Huang X Shi J Lu L Mental health considerations for children quarantined because of COVID-19. Lancet Child Adolesc Health. 2020;4:347-9. 10.1016/S2352-4642(20)30096-1 32224303 PMC7118598

[11] Qin Z Shi L Xue Y Lin H Zhang J Liang P Prevalence and Risk Factors Associated With Self-reported Psychological Distress Among Children and Adolescents During the COVID-19 Pandemic in China. JAMA Netw Open. 2021;4:e2035487. 10.1001/jamanetworkopen.2020.35487 33496797 PMC7838937

[12] Stickley A Koyanagi A Koposov R Blatný M Hrdlička M Schwab-Stone M Loneliness and its association with psychological and somatic health problems among Czech, Russian and U.S. adolescents. BMC Psychiatry. 2016;16:128. 10.1186/s12888-016-0829-2 27146137 PMC4857285

[13] Erzen E Çikrikci Ö The effect of loneliness on depression: A meta-analysis. Int J Soc Psychiatry. 2018;64:427-35. 10.1177/0020764018776349 29792097

[14] Lau S Chan DWK Lau PSY Facets of loneliness and depression among Chinese children and adolescents. J Soc Psychol. 1999;139:713-29. 10.1080/00224549909598251 10646306

[15] Brage D Campbell-Grossman C Dunkel J Psychological correlates of adolescent depression. J Child Adolesc Psychiatr Nurs. 1995;8:23-30. 10.1111/j.1744-6171.1995.tb00547.x 8630643

[16] Kumari A Singh P Impact of counseling on psychological health during lockdown of Covid-19. J Stat Manag Syst. 2020;24:53-65.

[17] Chang EC Wan L Li P Guo Y He J Gu Y Loneliness and Suicidal Risk in Young Adults: Does Believing in a Changeable Future Help Minimize Suicidal Risk Among the Lonely? J Psychol. 2017;151:453-63. 10.1080/00223980.2017.1314928 28486077

[18] Soklaridis S Lin E Lalani Y Rodak T Sockalingam S Mental health interventions and supports during COVID- 19 and other medical pandemics: A rapid systematic review of the evidence. Gen Hosp Psychiatry. 2020;66:133-46. 10.1016/j.genhosppsych.2020.08.007 32858431 PMC7442905

[19] Singh S Roy D Sinha K Parveen S Sharma G Joshi G Impact of COVID-19 and lockdown on mental health of children and adolescents: A narrative review with recommendations. Psychiatry Res. 2020;293:113429. 10.1016/j.psychres.2020.113429 32882598 PMC7444649

[20] Zhou X Snoswell CL Harding LE Bambling M Edirippulige S Bai X The Role of Telehealth in Reducing the Mental Health Burden from COVID-19. Telemed J E Health. 2020;26:377-9. 10.1089/tmj.2020.0068 32202977

[21] Li TMH Leung CSY Exploring student mental health and intention to use online counselling in Hong Kong during the COVID-19 pandemic. Psychiatry Clin Neurosci. 2020;74:564-5. 10.1111/pcn.13117 32686896 PMC7405056

[22] Schuster R Pokorny R Berger T Topooco N Laireiter AR The Advantages and Disadvantages of Online and Blended Therapy: Survey Study Amongst Licensed Psychotherapists in Austria. J Med Internet Res. 2018;20:e11007. 10.2196/11007 30563817 PMC6315274

[23] Sweeney GM Donovan CL March S Forbes Y Logging into therapy: Adolescent perceptions of online therapies for mental health problems. Internet Interv. 2016;15:93-9. 10.1016/j.invent.2016.12.001 30792959 PMC6371200

[24] Tylee A Haller DM Graham T Churchill R Sanci LA Youth-friendly primary-care services: how are we doing and what more needs to be done? Lancet. 2007;369:1565-73. 10.1016/S0140-6736(07)60371-7 17482988

[25] Christensen H Griffiths KM Korten A Web-based cognitive behavior therapy: Analysis of site usage and changes in depression and anxiety scores. J Med Internet Res. 2002;4:e3. 10.2196/jmir.4.1.e3 11956035 PMC1761927

